# The role of LHEP in spontaneous closure of full-thickness macular holes

**DOI:** 10.1007/s00417-025-07043-w

**Published:** 2025-12-04

**Authors:** B. Paschke, H. Helbig, M. A. Gamulescu

**Affiliations:** Universitätsklinikum Regensburg, Bavaria, Germany

**Keywords:** Lamellar-hole-associated epiretinal proliferation, Full-thickness macular hole, Epiretinal membrane, Vitreomacular traction, Spectral-domain optical coherence tomography

## Abstract

**Purpose:**

Spontaneous closure of full-thickness macular holes (SCMH) is a rare condition. However, this can sometimes be seen in patients waiting for operation. Here we present our morphologic findings in SCMH and discuss the possible pathogenesis.

**Methods:**

Retrospective analysis. Over the period of 2014 to 2024, 20 eyes of 20 patients showed SCMH. Morphologic characteristics were evaluated by SD-OCT imaging and patients were categorized by the existence of vitreomacular traction (VMT), epiretinal membrane (ERM) and lamellar hole associated epiretinal proliferation (LHEP). A relevant amount of epiretinal material of medium and homogenous reflectivity was labeled as LHEP. The study population consisted of 13 women and 7 men, all between ages of 52 and 85 years.

**Conclusions and importance:**

LHEP was identified in 12 of the 20 eyes (60%) with spontaneous hole closure. In 4 out of 20 eyes (20%) vitreomacular traction was found, 1 eye (5%) showed epiretinal membrane and another 3 (15%) no epiretinal pathologies at all. The frequent presence of LHEP in eyes with SCMH is in line with the recent postulations that LHEP might be the body’s attempt to counter the tangential traction on the retinal tissue around the hole and thus facilitate its closure. This understanding of the LHEP as a reparative process leads to the discussion whether a more conservative approach towards small full-thickness macular holes in association with LHEP may be justified.

**Key messages:**

***What is known*:**

- Spontaneous closure of macular holes is a rare phenomenon.

***What is new*:**

- LHEP is frequently present in eyes with spontaneous closure of macular holes.
- LHEP may be understood as a reparative process to facilitate hole closure.
- The presence of LHEP may have implications for the treatment of macular holes.

Spontaneous closure of full-thickness macular holes (SCMH) is a rare but recognized phenomenon, occasionally observed in patients awaiting operation. Here we present our morphologic findings in SCMH and discuss the possible pathophysiology.

In this retrospective analysis we reviewed 20 eyes of 20 patients that showed SCMH over the period of 2014 to 2024. None of the eyes had undergone vitreoretinal surgery prior to SCMH. Morphologic characteristics were evaluated by SD-OCT imaging and patients were categorized by the presence of vitreomacular traction (VMT), epiretinal membrane (ERM) and lamellar hole associated epiretinal proliferation (LHEP). A relevant amount of epiretinal material of medium and homogenous reflectivity was labeled as LHEP. The study population consisted of 13 women and 7 men, all between ages of 52 and 85 years.

LHEP was identified in 12 of the 20 eyes (60%) with spontaneous hole closure. In 4 out of 20 eyes (20%) vitreomacular traction was found prior to SCMH, 1 eye (5%) showed ERM and another 3 (15%) no epiretinal pathologies.

Although the term “lamellar hole-associated epiretinal proliferation” was initially used in the context of lamellar macular holes (LMH), similar epiretinal material has also been identified in full-thickness macular holes (FTMH), particularly those believed to evolve from LMH. The epiretinal tissue in these FTMH cases shares morphological and histological features with LHEP seen in LMH, such as homogenous reflectivity and close adherence to the inner retinal surface, appearing to bridge the gap between the edges of the hole. Consequently, the term LHEP has been adopted to describe this specific form of epiretinal proliferation in both LMH and certain FTMH cases, reflecting a presumed shared pathogenesis. Its incidence seems to be especially high if the posterior hyaloid is still attached to the macula [1, 2]. However, in our case series, no attachment of the posterior vitreous was found in LHEP patients. LHEP consists of proliferating cells on the epiretinal surface, which are presumed to originate from glial cells of the inner retina and is often associated with yellowish pigment around the hole [1, 2]. However, some studies suggest that hyalocytes and fibroblasts of the vitreous significantly contribute to the formation of LHEP [3]. The exact composition of LHEP remains uncertain.

In contrast to these features of LHEP, ERM not only contains glial cells but also transdifferentiated RPE cells and a more abundant extracellular matrix. Its idiopathic form is associated with the presence of PVD, while secondary ERM may develop following retinal tear, laser treatment, intraocular inflammation, or other retinal pathologies. Unlike LHEP, ERM can also exert tangential tractional forces, which are responsible for the characteristic retinal wrinkling seen on fundoscopy and visual symptoms like metamorphopsia.

The frequent presence of LHEP in SCMH supports the hypothesis that LHEP may represent a reparative cellular response aimed at counteracting tangential traction at the hole margins. This aligns with recent suggestions that LHEP could facilitate hole closure through intrinsic regenerative mechanisms [4].

Another point to consider is that the impact of LHEP on functional postoperative outcomes remains controversial. Several studies have reported that eyes with LHEP tend to have worse postoperative visual acuity compared to those without. This may be attributed to the disorganized proliferation of glial cells and Müller cell processes within the LHEP, which can lead to disruption of the foveal architecture and photoreceptor layer integrity [5].

The understanding of LHEP as a possible reparative process leads to the discussion of whether a more conservative approach may be justified in selected cases of small FTMH associated with LHEP.

Given the descriptive and retrospective nature of this study, caution is warranted in drawing definitive conclusions. However, this association between LHEP and SCMH, as well as its implication on potential treatment, invites further investigation.
